# Epigenetic age acceleration in adolescence: cross-sectional associations with dietary intake and prospective associations with cardiometabolic risk indicators in a Mexico City cohort

**DOI:** 10.1016/j.numecd.2026.104574

**Published:** 2026-01-23

**Authors:** Jennifer T. Lee, Jaclyn M. Goodrich, Dana C. Dolinoy, Karen E. Peterson, Martha M. Téllez-Rojo, Alejandra Cantoral, Libni A. Torres-Olascoaga, Maricruz Tolentino, Edward A. Ruiz-Narváez, Erica C. Jansen

**Affiliations:** a University of Michigan School of Public Health, Department of Nutritional Sciences, Ann Arbor, MI, USA; b University of Michigan School of Public Health, Department of Environmental Health Sciences, Ann Arbor, MI, USA; c National Institute of Public Health, Cuernavaca, Mexico; d Health Department, Iberoamericana University, Mexico City, Mexico; e Department of Nutrition, National Institute of Perinatology, Mexico City, 11000, Mexico; f University of Michigan, Sleep Disorders Center and Department of Neurology, Ann Arbor, MI, USA

**Keywords:** Dietary patterns, Nutrient intake, Epigenetic aging, Accelerated epigenetic age, Cardiometabolic risk factors, Adolescent

## Abstract

**Background and aims:**

To examine the cross-sectional relationship between dietary intake and epigenetic age acceleration, as well as the prospective relationship between epigenetic age acceleration and cardiometabolic parameters measured two years later.

**Methods and results:**

In 526 adolescents aged 7–18 years (average age 14.50) residing in Mexico City, dietary intake was assessed using a semi-quantitative food frequency questionnaire. Adherence to three dietary patterns was derived from principal component analysis. Blood leukocyte DNA methylation was measured with the Illumina Infinium MethylationEPIC BeadChip, from which epigenetic age acceleration was calculated for six epigenetic clocks: Horvath, Skin-Blood, PhenoAge, GrimAge, Pediatric-Buccal-Epigenetic (PedBE), and Wu. Nine cardiometabolic parameters were assessed two years after assessment of diet and epigenetic age acceleration. Linear regression models for sex-stratified associations were examined. Among males, the *Meat & Starchy foods* pattern was positively associated with Wu epigenetic age acceleration, showing a 0.096-year increase, while folate intake in females was associated with a 0.004-year decelerated GrimAge. Prospective analysis showed positive associations between epigenetic age acceleration and fat distribution and insulin resistance, particularly in males. In females, only GrimAge acceleration was associated in the expected manner with increased waist circumference (β = 0.62 cm), BMI (β = 0.25 kg/m^2^), fasting insulin (β = 0.86 μIU/mL), and insulin resistance (β = 0.21). Skin-Blood acceleration was associated with decreased HDL in males, and PedBE acceleration was associated with triglycerides in both sexes, though in opposing directions.

**Conclusion:**

Adolescent diet was not strongly associated with baseline epigenetic age acceleration. However, epigenetic age acceleration was associated prospectively with fat distribution and insulin resistance.

## 1. Introduction

Cardiometabolic disorders pose a significant global health challenge as the leading cause of mortality and morbidity worldwide [1]. Cardiometabolic risks escalate as individuals progress through life due to cumulative exposure to external risk factors, including those that occur as early as childhood [2].

Epigenetic age, a biomarker of biological aging based on epigenetic (i.e. DNA methylation) patterns, can reflect the cumulative influence of factors originating from lifestyle choices, environmental exposures, and genetic predisposition to cellular aging [3]. Several epigenetic clocks, including Horvath [4], Skin-Blood [5], PhenoAge [6], GrimAge [7], Pediatric-Buccal-Epigenetic (PedBE) [8], and Wu [9] have been developed, each capturing distinct aspects of biological aging. The Horvath and the Skin-Blood clocks are first-generation measures based on chronological aging, whereas the PhenoAge and GrimAge clocks incorporate clinical and mortality-related biomarkers to reflect health-related aging. The PedBE and Wu clocks are recently developed measures designed to estimate biological aging in children. Examining multiple clocks allows for a more comprehensive assessment of biological aging during adolescence. Epigenetic age acceleration, where epigenetic age exceeds chronological age, has been linked to increased risk of mortality, cognitive decline, and cardiometabolic disorders among adults [10], although results vary according to individual clocks. However, the extent to which epigenetic age acceleration might predict cardiometabolic risk factors earlier in life, potentially as early as adolescence, remains unclear.

Dietary patterns, such as those high in fruits, vegetables, and whole grains have been inversely associated with epigenetic aging [11], while those high in red meat, fast food, and sugar-sweetened beverages have been linked to accelerated epigenetic age [12]. These dietary patterns not only reflect the overall quality of the diet but also influence intake of methyl-donor nutrients, including folate, methionine, riboflavin, vitamin B6, and vitamin B12, which are essential in one-carbon metabolism, a process important for DNA methylation [13]. While previous studies have examined these relationships in adults [11,14] or early childhood [15,16], research during late childhood and adolescence remains sparse. Adolescence is an important developmental period characterized by rapid physiological, hormonal, and metabolic changes [17]. It is also when longer-term dietary behaviors can become established. Moreover, adolescence is a time when epigenetic changes can accelerate, making it a relevant period to study epigenetic aging.

Our study addresses this gap by examining, within a well-characterized cohort of Mexican adolescents, (1) the cross-sectional associations between dietary intake and multiple measures of epigenetic age acceleration, and (2) the prospective associations between baseline epigenetic age acceleration and cardiometabolic indicators two years later.

## 2. Methods

### 2.1. Study population

The study sample consisted of children from two of three sequentially enrolled birth cohorts of the Early Life Exposure in Mexico to ENvironmental Toxicants (ELEMENT) project [18]. Briefly, the initial recruitment of 1012 mother-child pairs occurred from 1997 to 2004 at prenatal clinics serving low- and middle-income populations within the Mexican Social Security Institute, Mexico City and were followed periodically. Longitudinal follow-up of the children was conducted at multiple time points from birth through childhood and adolescence. In 2015, data were collected from a subset of 554 adolescents undergoing pubertal transition (aged 9–18 years) at a research facility of the American British Cowdray (ABC) hospital in Mexico City. Baseline data on sociodemographic and health, dietary intake, and blood samples were collected. A follow-up visit was conducted approximately 2 years later (average time difference 1.98, SD 0.45). Anthropometric assessments, 8-h fasting blood samples, and interview-based questionnaires were collected from the children during the in-person visits. Data from the 2015 baseline visit and the subsequent follow-up visit were used in this study. The final analysis included 526 adolescents with complete data on dietary intake, and at least one of six epigenetic age measures – Horvath [4], Skin-Blood [5], PhenoAge [6], GrimAge [7], PedBE [8], and Wu clocks [9] – or one of nine cardiometabolic risk indicators. For the prospective analysis, data were available for 493 adolescents on body mass index (BMI), for 492 adolescents on waist circumference, systolic and diastolic blood pressure, and 367 adolescents for fasting glucose, fasting triglycerides, fasting high-density lipoprotein (HDL) cholesterol, fasting insulin, and Homeostatic Model Assessment of Insulin Resistance (HOMA-IR). The research protocols were approved by institutional review boards at the Mexico National Institute of Public Health, the ABC Hospital, and the University of Michigan. Informed consent was obtained from the parents of all adolescents, with additional assent obtained from the adolescents themselves.

### 2.2. Dietary intake

As previously described [19], a validated semi-quantitative 116-item food frequency questionnaire (FFQ) adapted from the 2006 Mexican National Health and Nutrition Survey was used to assess habitual dietary intake over the previous week [20]. Age-specific versions of the FFQ were administered by trained interviewers to children 7–11 years and adolescents aged 12 years or older to assess frequency and portion size of food items consumed over the past week. Nine response options, ranging from “never” to “≥6 times per day,” were provided. Subsequently, raw responses were converted into daily serving equivalents. Estimated daily intake of energy (kcal) and methyl-donor nutrients (folate, methionine, riboflavin, vitamin B6, and vitamin B12) was calculated by multiplying the nutrient contents in each food item with frequency of usual intake and taking the sum of each nutrient across all food items using a software developed by the National Institute of Public Health [21]. Food items were classified into 35 distinct food groups based on nutritional similarity and cultural relevance. Each food group underwent adjustment for total energy intake using the residual method. Principal component analysis (PCA) was utilized to generate continuous dietary pattern scores, with higher scores reflecting a stronger alignment with the corresponding dietary pattern [19]. Briefly, the *Plant-based & lean proteins* pattern indicated a higher frequency of consumption of vegetables, fruits, soup, fish, water, and unsweetened beverages. The *Meat & starchy* pattern was characterized by a higher frequency of intake of processed foods (“Western” style), including chips, refined grains, sugary drinks, processed meats, high-fat dairy products, Mexican staples (tacos, quesadillas), potatoes, fried plantains, soup, legumes, and corn tortillas. Finally, the *Eggs, milk & refined grain* (i.e. *Breakfast*) pattern identified a higher frequency of consumption of refined grains, milk, sweetened milk products, mayonnaise/margarine, and eggs. Dietary methyl-donor nutrient intakes were also adjusted for total energy intake using the residual method.

### 2.3. Epigenetic age

Six epigenetic age measures (Horvath, Skin-Blood, PhenoAge, GrimAge, PedBE, and Wu) were derived using DNA methylation data from age-related CpG sites across the genome and different tissues as described in prior studies [22]. Whole blood was collected through venipuncture in ethylenediaminetetraacetic acid (EDTA) tubes and stored at − 80 °C prior to processing. DNA was extracted from blood leukocytes using the Flexigene kit (Qiagen). Subsequently, sodium bisulfite treatment was applied to convert unmethylated cytosines to uracils, while methylated cytosines remained unaffected [23]. DNA methylation levels across 850,000 cytosine-guanine dinucleotides were assessed using the Infinium MethylationEPIC BeadChip (Illumina) [24]. Based on the raw image files, average methylation levels per CpG site (beta values) were obtained from the R statistical environment using the *minfi* package [25]. Background correction was performed using the Noob method, dye bias was adjusted with RELIC, and quantile normalization was applied to ensure comparability across samples [26, 27]. Quality control involved eliminating those with coverage of <3 beads at more than 5 % of CpG sites, those exceeding 5 % failed probes based on the background comparison threshold, those outside the acceptable bisulfite conversion rate, those with outlier DNA methylation distributions across all CpG sites, and those with inconsistent estimated sex. All samples passed the quality control check.

The New Methylation Age Calculator (https://dnamage.genetics.ucla.edu/new ) was used to process the Infinium methylation data to generate several epigenetic age clocks: the original Horvath clock, which was designed to be applicable across different tissue types [4]; the Skin-Blood clock, which provides a more accurate estimate for DNA from skin, blood, or saliva [5]; and PhenoAge and GrimAge, two composite epigenetic clocks that are regarded as superior predictors of all-cause mortality, cancers, and other adverse health conditions compared to the original clocks [6,7]. Two epigenetic clocks were estimated for children, the PedBE and the Wu clocks, using the *methyl-clock* package in R [28]. Given that these epigenetic clocks were trained on distinct sets of CpG sites, consistency was achieved by utilizing the same number of CpG sites originally identified and validated for their robust correlations with chronological age or age-related pathologies within the corresponding initial studies. Epigenetic age acceleration was obtained as the residual from a linear regression model regressing epigenetic age clocks on chronological age. These residuals reflect the extent of epigenetic age acceleration or deceleration, where positive values indicate accelerated epigenetic or biological aging and negative values signify decelerated aging relative to chronological age.

### 2.4. Cardiometabolic parameters

Duplicate measurements were collected by trained research staff during the follow-up visit. Body weight was measured to the nearest 0.1 kg using a digital scale (BAME Model 420; Catálogo Médico) and InBody 230 (Biospace Co, Ltd, Seoul, Republic of Korea). Height was measured to the nearest 0.5 cm, and waist circumference was measured to the nearest 0.1 cm using the non-stretchable measuring tape SECA (model 201, Hamburg, Germany) [29]. Two sets of readings for both systolic and diastolic blood pressure were taken while the participant was seated, using an automated blood pressure monitor (BPM-200 Medical Devices Blood Pressure Monitor, BpTRU; Coquitlam, BC, Canada). A range of cuffs was available during the study visit, including those suitable for children (13–18 cm), small adults (18–26 cm), regular adults (26–34 cm), large adults (32–43 cm), and extra-large adults (41–52 cm). Staff members ensured the proper selection of cuffs based on the participant’s arm dimensions. The average of the two measurements was used in the analysis.

Fasting blood samples were utilized to assess serum glucose levels using automated chemiluminescence immunoassay (Immulite^®^ 1000; Siemens Medical Solutions, Erlangen, Germany) [30]. Triglyceride and HDL cholesterol levels were analyzed using a biochemical analyzer (Cobas Mira Plus; Roche Diagnostics) [30]. Fasting serum insulin was determined through an enzyme-linked immunosorbent assay chemiluminescence method with Immulite^®^ 1000; Siemens Medical Solutions, Erlangen, Germany [29]. HOMA-IR was computed [fasting plasma glucose (mmol/L) × fasting serum insulin (mU/L))/22.5], with higher values indicating lower insulin sensitivity or insulin resistance.

### 2.5. Covariates

During the baseline visit, interview questions collected information on continuous age in years, sex (categorized as male or female), ever tried smoking, and household socioeconomic status (SES). Household SES was assessed from a validated 13-item questionnaire on housing quality, services, and education of the head of household, as administered by the AMAI (Asociacíon Mexicana de Agencias de Investigación de Mercados y Opinión Pública, version 13x6) based on the results of the National Survey of Household Income and Expenditure 2005 in Mexico [31]. We classified this scale into four categories: upper (A/B), upper middle or middle (C+/C), lower middle or lower (D+/D), and lowest (E) class. Self-reported ever tried smoking was assessed from the question: “Have you ever smoked in your life, even a single puff?” (yes, no, don’t know).

Other covariates examined in this analysis were maternal education, physical activity, sedentary behavior, pubertal status, ever consumed alcohol, and average age difference between the two in-person visits. Maternal education was reported by mothers at enrollment and was categorized into four categories: none or elementary school, middle school, commercial/technical career or high school, and undergraduate or graduate degree. Average duration of sedentary behavior and physical activity (minutes per day) was estimated from 7-day wrist accelerometer data as previously described [9]. Activity levels were categorized as sedentary, light, moderate, and vigorous according to the validated Chandler’s Vector Magnitude cutoffs [32]. The total minutes spent in activities as moderate or vigorous intensity were summed to calculate moderate-to-vigorous physical activity. Pubertal status was assessed separately for boys and girls by trained physicians during the in-person visit: for girls, by the onset of menarche, and for boys, by testicular volume measured with orchidometers (ranged from 1 to 25 mL). Pubertal status was then classified dichotomously as “early” or “late” stage, with a “late” pubertal stage defined as having experienced menarche for girls or having a testicular volume of ≥15 mL for boys. Self-reported ever tried alcohol was determined from the question “Have you ever in your life consumed any alcoholic drink or substance?” (yes, no, don’t know). The age difference between the two in-person visits was calculated by subtracting the participant’s age at the 2015 in-person visit from their age at the subsequent visit.

Additional covariates included batch effects and cell type proportions. Five variables representing DNA methylation data batch effects, identified through principal component analysis of data from control probes, were considered. We also evaluated estimates of cell type composition, which included proportions of monocytes, granulocytes, natural killer cells, CD8 T cells, CD4 T cells, and B cells, and relative abundance of memory and effector T cells, plasma-blasts, naïve CD8 T cells, and naïve CD4 T cells.

### 2.6. Statistical analysis

Spearman’s correlation coefficients were used to identify potential confounders and examine the linear relationships between covariates of interest, dietary patterns and methyl-donor nutrient intakes, epigenetic age measures, and cardiometabolic risk indicators (Supplemental Table 1). The selection of covariates was further informed by considerations of biological plausibility and potential for multicollinearity. Chi-square tests for categorical variables and simple linear regression for continuous variables were used to compare participant characteristics by sex. Linear regression analyses were conducted to investigate (1) the cross-sectional associations between eight dietary intake variables (three dietary patterns and five methyl-donor nutrient intakes) and six measures of epigenetic age acceleration (Horvath, Skin-Blood, PhenoAge, GrimAge, PedBE, and Wu), and (2) the prospective associations between epigenetic age acceleration and nine cardiometabolic markers of adiposity and fat distribution (BMI and waist circumference), hypertension (systolic and diastolic blood pressure), insulin resistance (glucose, insulin, and HOMA-IR), and dyslipidemia (triglycerides and HDL). All models were adjusted for age, sex, household SES, ever tried smoking, batch effects, and estimated proportions of monocytes, granulocytes, natural killer cells, CD8^+^ T lymphocytes, CD4^+^ T lymphocytes, plasma blasts, memory and effector T cells, naïve CD8 T cells, and naïve CD4 T cells within the samples. Sex-stratified analyses were performed. Sensitivity analysis included: 1) removal of age from the adjusted model, 2) additional adjustment for maternal education, physical activity, sedentary behavior, pubertal status, alcohol drinking, height, and average age difference between two in-person visits, and 3) multivariate regression models of epigenetic age acceleration and jointly-considered cardiometabolic risk indicators. In cases where categorical covariates had missing values, participants were included in the analysis as a separate category within the variable while a complete case approach was used for missing physical activity and sedentary behavior data. No missing values were observed for other continuous covariates. Results used a significance threshold of *p-value<0.05*. To account for multiple testing across epigenetic age acceleration measures, Bonferroni correction was applied for the six epigenetic clocks, with statistical significance defined as *α*=*0.05/6*=*0.0083*. Statistical analyses were conducted using R-Statistics (version 4.3.2, R Foundation for Statistical Computing, Vienna, Austria).

## 3. Results

The study population included 526 adolescents (Table 1). The mean chronological age was 14.50 ± 2.12 years with 279 (53.04 %) females. No significant sex differences were observed for age, household SES, ever tried smoking, ever tried alcohol, maternal education, or physical activity. More female adolescents were in the later stage of puberty compared to males (*p<0.001*). Males had a higher duration of sedentary behavior than females (*p*=*0.021*).

The measures of epigenetic age acceleration were moderately positively correlated with one another, although GrimAge had no correlation with either of the pediatric clocks (Wu and PedBE; Supplemental Table 1). Dietary patterns and methyl-donor nutrient intakes were not significantly associated with epigenetic age acceleration, except in sex-stratified models (Table 2). Specifically, among males, adherence to the *Meat & starchy foods* pattern was positively associated with 0.10-year Wu clock acceleration [standard error (SE) = 0.03, *p*=*0.006*]. In females, dietary folate intake was positively associated with 4.29 × 10^−3^-year GrimAge clock deceleration (SE = 2.09 × 10^−3^, *p*=*0.041*). After Bonferroni correction, no associations in the primary analysis were statistically significant, while the male-specific association with the Wu clock remained statistically significant.

**Fig. 1.**
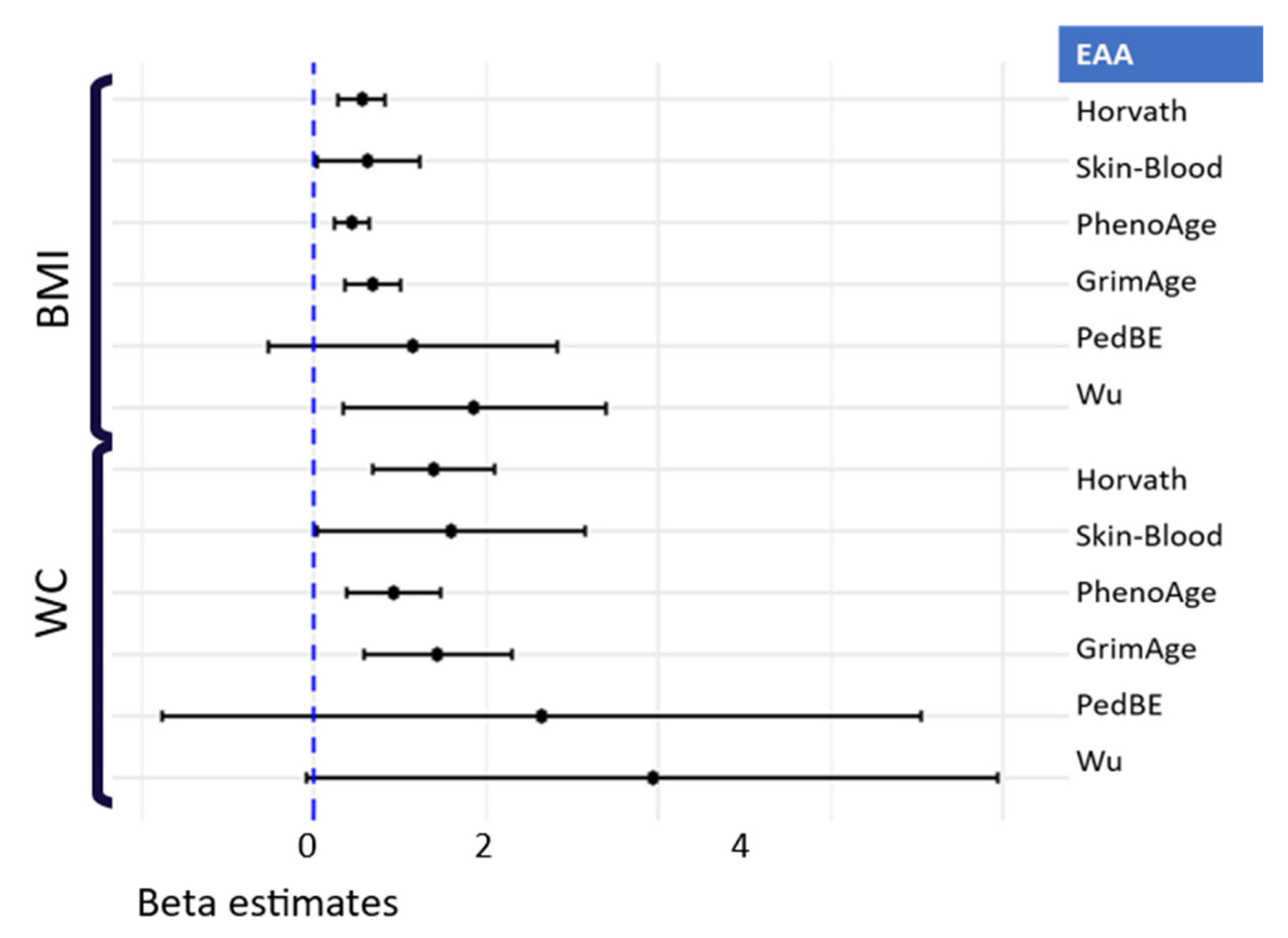
Associations between EEAA and selected fat distribution measures.

**Fig. 2.**
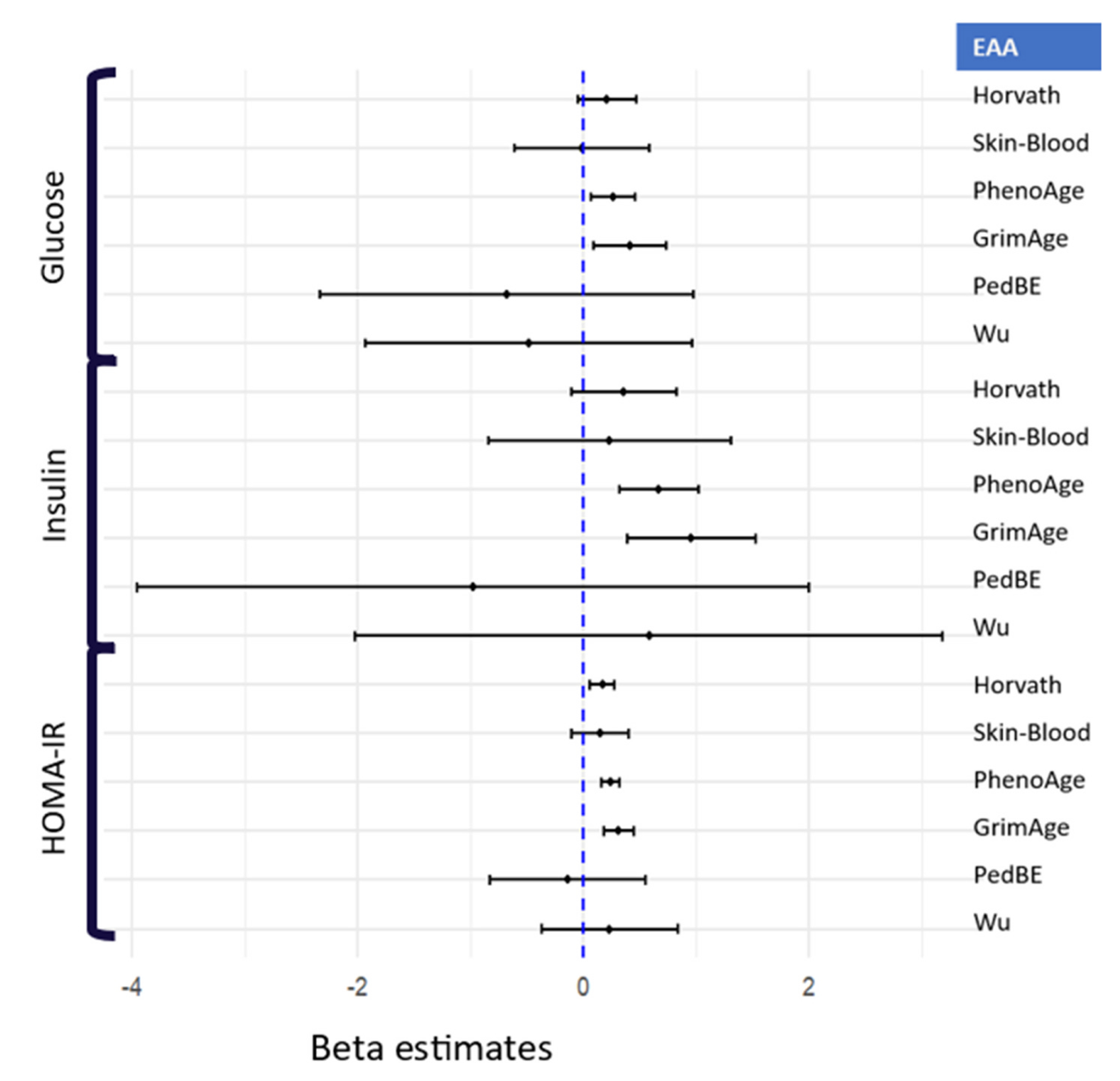
Associations between EEAA and insulin sensitivity measures.

In the prospective analysis of epigenetic age acceleration and cardiometabolic markers assessed two years later, several significant associations were observed (Table 3). Horvath, PhenoAge, and GrimAge accelerations were positively associated with measures of adiposity and fat distribution (Fig. 1). Additionally, significant positive associations were found between PhenoAge and GrimAge accelerations with fasting insulin and HOMA-IR (Fig. 2). After Bonferroni correction, most associations remained statistically significant, although associations with BMI became non-significant for all clocks except GrimAge, and the association between PhenoAge acceleration and waist circumference was no longer significant.

Sex-stratified analysis showed that associations between epigenetic age acceleration and measures of adiposity and fat distribution. were more apparent among males (Table 4). For example, Horvath acceleration was associated with a 0.94 cm increase in waist circumference (SE = 0.26, *p*<*0.001*) and a 0.28 kg/m^2^ increase in BMI (SE = 0.09, *p*=*0.003*), while PhenoAge showed smaller effect estimates (0.64 cm, SE = 0.21, *p*=*0.002*; 0.22 kg/m^2^, SE = 0.07, *p*=*0.003*) in males. In females, only GrimAge acceleration was associated in the expected manner with increased waist circumference (β = 0.62 cm), BMI (β = 0.25 kg/m^2^), fasting insulin (β = 0.86μIU/mL), and insulin resistance (β = 0.21). Skin-Blood acceleration was associated with decreased HDL in males, and PedBE acceleration was associated with triglycerides in both sexes, though in opposing directions (β = 23.34, *p*=*0.001* for males and β = − 21.57, *p*=*0.007* for females). After Bonferroni correction, several sex-specific associations remained statistically significant, including Horvath and PhenoAge accelerations with adiposity and insulin-related measures in males, and PedBE acceleration with triglycerides in females.

A sensitivity analysis that utilized a multivariate regression model to account for the correlation of cardiometabolic risk outcomes did not result in appreciable differences. Similarly, the removal of age from the adjusted model did not alter the estimates. Sensitivity analysis that additionally adjusted for maternal education, physical activity, sedentary behavior, pubertal status, alcohol drinking, height, and average age difference between two in-person visits showed minor attenuations in the effect estimates but no changes to the overall interpretations (Supplemental Table 2). For example, Horvath acceleration was associated with a 0.53 cm increase in waist circumference (SE = 0.18, *p*=*0.003*) and a 0.16 increase in BMI (SE = 0.07, *p*=*0.025*) whereas the model with fewer confounders yielded estimates of 0.60 (SE = 0.18, p = 0.001) and 0.18 (SE = 0.07, p = 0.008), respectively.

## 4. Discussion

This study showed no significant associations between dietary intake and epigenetic age acceleration overall; however, our findings suggested a few sex-specific relationships. Our findings also indicated positive relationships between epigenetic age acceleration in adolescence and cardiometabolic risk indicators measured two years later, particularly measures of adiposity and insulin resistance. Sex-stratified analyses revealed that these associations were more consistently observed in males.

The lack of statistically significant results in our cross-sectional assessment of diet and epigenetic age acceleration may be due to inadequately capturing the critical period of exposure necessary to observe significant effects on epigenetic age. Another possibility for the lack of significant associations may stem from insufficient variability in diet to differentiate between higher and lower adherence to dietary patterns or nutrient intake [19]. The dietary patterns observed in this population may also not be reflective of longer-term dietary patterns, and sustained exposure over many years may be required to manifest detectable alterations in epigenetic aging [14]. Nonetheless, our sex-specific results on diet and epigenetic aging measures are consistent with a previous intervention that showed potential sex-specific differences [33] and may indicate a role for sex hormones. Estrogen may be protective against oxidative stress and inflammation implicated in biological aging processes [34]. Estrogen also influences visceral fat distribution, thereby potentially decelerating epigenetic aging in females in response to dietary factors [34].

The null findings between diet and epigenetic aging measures during adolescence could also be life stage-specific. Most existing research examining the relationship between dietary intake and epigenetic age acceleration has been conducted in adults, with studies showing that higher diet quality is associated with decelerated epigenetic aging [11,14], although findings regarding nutrient intake have been mixed and unclear [35–37]. Findings are a little more consistent during fetal development and early childhood, when nutritional exposures have been shown to impact epigenetic changes [38]. Much less research has focused on the adolescent period, which is characterized by rapid growth, hormonal changes, and metabolic adjustments. During this time, dietary intake may interact with epigenetic mechanisms differently compared to other lifestages [39]. An additional factor to consider is that adolescents progress through puberty at different rates, and associations may vary according to differences in the pubertal process itself. We were not powered to conduct such analyses, so future work is needed to test this hypothesis.

In contrast to the associations with diet, the associations between epigenetic aging acceleration and cardiometabolic outcomes were much more robust, especially for adiposity and fat distribution and insulin resistance measures. Epigenetic age acceleration has been positively associated with insulin resistance in previous cross-sectional analyses of adolescents [40]. Moreover, the observed association between the Horvath clock and later adiposity indicators suggests that the age-related biological process may influence subsequent obesity risk [4,5]. Since GrimAge and PhenoAge are second generation clocks trained to estimate disease and mortality risk [6,7], our findings suggest that individuals with increased biological age as indicated by these clocks are at an increased risk of developing obesity and insulin resistance over time, and that this process may be underway during the adolescence. These epigenetic clocks may be predictive of future obesity and potentially serve as early indicators for adverse cardiometabolic health. Future research of these epigenetic clocks should include longer-term sex-stratified investigations of weight gain and body composition (e.g., muscle mass vs fat mass) from adolescence to young adulthood.

A key strength of this research is its focus on Mexican adolescents, a relatively underrepresented population in the context of dietary intake and epigenetic age acceleration. However, the focus on Mexican adolescents may limit the generalizability of the findings. The FFQ only assessed dietary intake over the previous week, which may not accurately capture long-term, habitual dietary patterns, potentially leading to recall bias or an underrepresentation of consistent dietary behaviors. Moreover, there could be unmeasured confounders of dietary intake in this analysis, including family income. Although we had a measure of socioeconomic status, we did not evaluate household income directly. Additionally, the cross-sectional design of the relationship between dietary intake and epigenetic age acceleration might not fully capture the impact of diet on epigenetic age, potentially introducing reverse causality. Future research should address this limitation by examining how dietary intake may impact epigenetic age over time and explore how epigenetic age may respond to changes in dietary intake. Another limitation is that several epigenetic clocks used in this study may not be fully optimized for pediatric populations, particularly during adolescence. Finally, the significant relationships observed may be influenced by multiple comparisons, which could limit the robustness of the findings.

While our findings indicated no direct link between dietary intake and epigenetic age acceleration, the observed associations between accelerated epigenetic age and subsequent measures of fat distribution and insulin resistance suggest the potential of using epigenetic age in mid-to-late adolescence as a predictive marker for future cardiometabolic risk. There is a need for research into modifiable factors influencing the epigenetic modification of the biological aging process in adolescents, and on the role epigenetic aging may play on long-term cardiometabolic health outcomes.

## Supplementary Material

Supplemental Table 1

Supplemental Table 2

## Figures and Tables

**Table 1:** Study participant characteristics.

Characteristics^+^	All (n = 526)	Male (n = 247)	Female (n = 279)	*p*-value
Age (years)	14.50 (2.12)	14.50 (2.05)	14.49 (2.18)	*0.946*
Age at follow-up	16.45 (2.13)	16.43 (2.02)	16.47 (2.22)	*0.839*
Household socioeconomic status				0.272
Upper class	34 (6.50)	20 (8.10)	14 (5.02)	
Upper middle or middle class	249 (47.30)	121 (48.99)	128 (45.88)	
Lower middle or low class	189 (35.90)	85 (34.41)	104 (37.28)	
Lowest class	54 (10.30)	21 (8.50)	33 (11.83)	
Ever smoking				0.591
Yes	132 (25.10)	67 (27.13)	65 (23.30)	
No	390 (74.10)	178 (72.06)	212 (75.99)	
Missing	4 (0.80)	2 (0.81)	2 (0.72)	
Ever alcohol drinking				0.493
Yes	402 (76.40)	183 (74.09)	219 (78.49)	
No	120 (22.80)	62 (25.10)	58 (20.79)	
Missing	4 (0.80)	2 (0.81)	2 (0.72)	
Maternal education				0.203
None or elementary	44 (8.40)	14 (5.67)	30 (10.75)	
Middle school	177 (33.70)	82 (33.20)	95 (34.05)	
Technical or high school	205 (39.00)	102 (41.30)	103 (36.92)	
College or higher	99 (18.80)	48 (19.43)	51 (18.28)	
Missing	1 (0.20)	1 (0.40)	0 (0.00)	
Pubertal status				*<0.001* *
Early stage	114 (21.70)	72 (29.15)	42 (15.05)	
Later stage	399 (75.90)	163 (65.99)	236 (84.59)	
Missing	13 (2.50)	12 (4.86)	1 (0.36)	
MVPA (hours/day)	1.31 (0.48)	1.29 (0.50)	1.33 (0.45)	*0.315*
Sedentary (hours/day)	10.01 (1.22)	10.14 (1.23)	9.89 (1.21)	*0.021* *
Energy (kcal) intake	2272.77 (874.30)	2523.29 (866.76)	2050.99 (820.77)	*<0.001* *
Dietary intake				
*Plant-based & lean protein* pattern	1.79 (1.01)	1.83 (1.10)	1.75 (0.91)	*0.408*
*Meat & starchy foods* pattern	2.09 (1.01)	2.14 (0.98)	2.05 (1.03)	*0.288*
*Breakfast* pattern	2.71 (0.99)	2.75 (0.94)	2.67 (1.03)	*0.359*
Folate intake (mcg DFE)	247.35 (66.98)	242.63 (61.60)	251.53 (71.26)	*0.128*
Methionine intake (g)	1.28 (0.40)	1.30 (0.46)	1.26 (0.34)	*0.337*
Riboflavin intake (mg)	1.33 (0.39)	1.31 (0.39)	1.35 (0.39)	*0.267*
Vitamin B6 intake (mg)	1.55 (0.44)	1.57 (0.46)	1.54 (0.42)	*0.592*
Vitamin B12 intake (mcg)	3.75 (2.19)	3.81 (2.67)	3.69 (1.66)	*0.547*
Epigenetic age measure				
Horvath	17.55 (4.19)	18.23 (4.10)	16.94 (4.19)	*<0.001* *
Skin-Blood	10.51 (2.41)	10.46 (2.34)	10.55 (2.47)	*0.675*
PhenoAge	2.79 (5.99)	2.08 (5.47)	3.42 (6.37)	*0.011* *
GrimAge	21.72 (3.47)	21.89 (3.17)	21.56 (3.72)	*0.283*
PedBE	7.33 (0.63)	7.37 (0.58)	7.29 (0.66)	*0.189*
Wu	8.36 (0.76)	8.41 (0.77)	8.32 (0.76)	*0.192*
Epigenetic age acceleration				
Horvath	0.08 (3.46)	0.75 (3.43)	−0.51 (3.38)	*<0.001* *
Skin-Blood	−0.01 (1.43)	−0.06 (1.45)	0.04 (1.40)	*0.436*
PhenoAge	−0.34 (4.69)	−1.06 (4.37)	0.30 (4.88)	*0.001* *
GrimAge	0.20 (2.62)	0.37 (2.46)	0.05 (2.74)	*0.157*
PedBE	−0.07 (0.56)	−0.03 (0.52)	−0.10 (0.59)	*0.147*
Wu	0.03 (0.58)	0.08 (0.57)	−0.01 (0.59)	*0.087*

SD=Standard deviation; MVPA =Moderate and vigorous physical activity; BMI=Body mass index; WC=Waist circumference; BP=Blood pressure; HDL=High-density lipoprotein; HOMA-IR=Homeostasis Model Assessment of Insulin Resistance; n = 491 with physical activity variables; n = 508 with sleep variables; n = 524 with epigenetic age acceleration measures; n = 490 with body mass index; n = 489 with systolic and diastolic blood pressure and waist circumference; n = 367 with fasting glucose, fasting insulin, HOMA-IR, triglycerides and HDL measures; Chi-square test (categorical variables) and simple linear regression (continuous variables) were used to compare participant characteristics between sex.

* Statistically significance at *p-value<0.05*.

+ Participant characteristics expressed as mean (SD) or n (%).

**Table 2:** Associations between dietary intake and epigenetic age acceleration, all and stratified by sex.

	Horvath		Skin-Blood		PhenoAge		GrimAge		PedBE		Wu	
	Beta (SE)^a^	*p*	Beta (SE)^a^	*p*	Beta (SE)^a^	*p*	Beta (SE)^a^	*p*	Beta (SE)^a^	*p*	Beta (SE)^a^	*p*
*Plant-based & lean proteins* ^b^												
All	−0.111 (0.127)	*0.382*	−0.022 (0.058)	*0.712*	−0.254 (0.168)	*0.131*	−0.080 (0.106)	*0.448*	−0.011 (0.021)	*0.598*	0.021 (0.023)	*0.356*
Male	−0.127 (0.176)	*0.470*	0.030 (0.080)	*0.709*	−0.243 (0.224)	*0.280*	−0.005 (0.136)	*0.972*	−0.022 (0.027)	*0.405*	0.039 (0.031)	*0.198*
Female	−0.080 (0.190)	*0.675*	−0.087 (0.091)	*0.340*	−0.356 (0.262)	*0.176*	−0.215 (0.168)	*0.203*	0.001 (0.033)	*0.967*	0.019 (0.035)	*0.588*
*Meat & starchy foods* ^b^												
All	−0.003 (0.126)	*0.980*	0.018 (0.058)	*0.757*	0.101 (0.166)	*0.542*	0.008 (0.104)	*0.939*	0.001 (0.020)	*0.977*	0.024 (0.023)	*0.286*
Male	−0.012 (0.201)	*0.954*	0.156 (0.090)	*0.085*	0.440 (0.255)	*0.086*	−0.115 (0.156)	*0.461*	−0.035 (0.030)	*0.245*	0.096 (0.034)	*0.006* * ^+^
Female	0.010 (0.165)	*0.952*	−0.102 (0.079)	*0.196*	−0.192 (0.228)	*0.401*	0.060 (0.146)	*0.682*	0.012 (0.029)	*0.665*	−0.015 (0.030)	*0.621*
*Breakfast* ^b^												
All	−0.119 (0.128)	*0.355*	−0.070 (0.059)	*0.232*	0.044 (0.170)	*0.798*	−0.026 (0.107)	*0.805*	0.007 (0.021)	*0.730*	−0.031 (0.023)	*0.171*
Male	−0.085 (0.207)	*0.681*	−0.137 (0.093)	*0.141*	0.133 (0.264)	*0.615*	−0.147 (0.160)	*0.358*	−0.005 (0.031)	*0.868*	−0.020 (0.036)	*0.570*
Female	−0.168 (0.165)	*0.309*	−0.034 (0.079)	*0.669*	−0.086 (0.228)	*0.706*	0.010 (0.146)	*0.943*	0.015 (0.029)	*0.602*	−0.035 (0.030)	*0.243*
*Folate intake*												
All	−1.95 × 10^−3^ (1.91 × 10−3)	*0.307*	−4.25 × 10^−4^ (8.75 ×10−4)	*0.627*	−2.83 × 10^−3^ (2.52 × 10−3)	*0.262*	−2.52 × 10^−3^ (1.58 × 10−3)	*0.111*	2.32 × 10^−4^ (3.08 × 10−4)	*0.452*	2.61 × 10^−5^ (3.43 × 10−4)	*0.939*
Male	−2.30 × 10^−3^ (3.14 × 10^−3^)	*0.464*	2.93 × 10^−4^ (1.42 × 10^−3^)	*0.837*	−3.89 × 10^−3^ (4.01 × 10^−3^)	*0.332*	6.82 × 10^−4^ (2.43 × 10^−3^)	*0.780*	3.27 × 10^−4^ (4.76 × 10^−4^)	*0.492*	3.20 × 10^−4^ (5.46 × 10^−4^)	*0.558*
Female	−1.56 × 10^−3^ (2.38 × 10^−3^)	*0.513*	−8.71 × 10^−4^ (1.14 × 10^−3^)	*0.447*	−2.23 × 10^−3^ (3.30 × 10^−3^)	*0.499*	−4.29 × 10^−3^ (2.09 × 10^−3^)	*0.041* *	1.74 × 10^−4^ (4.13 × 10^−4^)	*0.673*	−1.06 × 10^−4^ (4.40 × 10^−4^)	*0.809*
*Methionine intake*												
All	−0.430 (0.321)	*0.181*	0.004 (0.147)	*0.980*	0.002 (0.425)	*0.997*	0.043 (0.267)	*0.871*	0.066 (0.052)	*0.206*	−0.011 (0.058)	*0.855*
Male	−0.605 (0.423)	*0.153*	−0.184 (0.191)	*0.339*	0.015 (0.542)	*0.979*	0.072 (0.329)	*0.828*	0.103 (0.064)	*0.109*	−0.082 (0.074)	*0.264*
Female	−0.475 (0.517)	*0.359*	0.293 (0.248)	*0.238*	−0.378 (0.716)	*0.598*	−0.094 (0.458)	*0.838*	0.004 (0.090)	*0.964*	0.053 (0.095)	*0.580*
*Riboflavin intake*												
All	−0.122 (0.328)	*0.710*	0.068 (0.150)	*0.652*	−0.333 (0.434)	*0.443*	−0.041 (0.273)	*0.880*	−0.008 (0.053)	*0.882*	−0.048 (0.059)	*0.417*
Male	−0.814 (0.492)	*0.100*	−0.064 (0.224)	*0.773*	−0.713 (0.631)	*0.260*	0.101 (0.384)	*0.793*	−0.038 (0.075)	*0.618*	−0.115 (0.086)	*0.182*
Female	0.344 (0.446)	*0.441*	0.160 (0.214)	*0.456*	−0.301 (0.617)	*0.626*	−0.282 (0.394)	*0.474*	0.009 (0.077)	*0.903*	0.010 (0.082)	*0.901*
*Vitamin B6 intake*												
All	−0.405 (0.287)	*0.159*	−0.055 (0.132)	*0.676*	0.110 (0.381)	*0.772*	−0.154 (0.239)	*0.519*	−0.003 (0.046)	*0.957*	0.038 (0.052)	*0.461*
Male	−0.303 (0.413)	*0.464*	0.074 (0.187)	*0.694*	0.193 (0.529)	*0.715*	0.260 (0.320)	*0.417*	−0.024 (0.063)	*0.706*	0.061 (0.072)	*0.393*
Female	−0.403 (0.402)	*0.317*	−0.176 (0.193)	*0.361*	0.172 (0.557)	*0.758*	−0.413 (0.355)	*0.246*	0.004 (0.070)	*0.949*	−0.001 (0.074)	*0.988*
*Vitamin B12 intake*												
All	−0.091 (0.059)	*0.122*	−0.004 (0.027)	*0.878*	−0.013 (0.078)	*0.864*	−0.016 (0.049)	*0.747*	0.012 (0.009)	*0.198*	−0.003 (0.011)	*0.769*
Male	−0.138 (0.074)	*0.063*	−0.031 (0.034)	*0.361*	0.034 (0.095)	*0.719*	0.015 (0.058)	*0.796*	0.017 (0.011)	*0.122*	−0.012 (0.013)	*0.363*
Female	−0.026 (0.104)	*0.800*	0.056 (0.050)	*0.265*	−0.144 (0.144)	*0.319*	−0.075 (0.092)	*0.414*	−0.002 (0.018)	*0.909*	−0.001 (0.019)	*0.954*

SE=Standard error; BMI=Body mass index; Epigenetic age acceleration reported in years.

* statistically significant at *p<0.05.*

+ statistically significant at Bonferroni corrected *p=0.0083(0.05/6)*.

a Model adjusted for sex, socioeconomic status, ever smoking, batch effects, and cell types.

b Dietary patterns derived from principal component analysis.

**Table 3:** Associations between epigenetic age acceleration and cardiometabolic risk indicators at follow-up.

	BMI, kg/m^2^	WC, cm	Systolic BP, mmHg	Diastolic BP, mmHg	Triglycerides, mg/dL	HDL, mg/dL	Glucose, mg/dL	Insulin, mU/mL	HOMA IR
	Beta(SE)	Beta(SE)	Beta(SE)	Beta(SE)	Beta(SE)	Beta(SE)	Beta(SE)	Beta(SE)	Beta(SE)
Horvath clock	0.18 (0.07)	0.60 (0.18)	−0.05 (0.15)	−0.09 (0.11)	0.50 (0.95)	−0.30(0.16)	0.11 (0.13)	0.26 (0.24)	0.07 (0.05)
*p-value*	*0.008* *	*<0.001* * ^+^	*0.732*	*0.400*	*0.597*	*0.068*	*0.404*	*0.273*	*0.237*
Skin-Blood	0.22 (0.15)	0.70 (0.40)	0.01 (0.33)	−0.03 (0.25)	0.25 (2.20)	−0.63(0.38)	−0.12 (0.31)	0.13 (0.55)	0.05 (0.13)
*p-value*	*0.146*	*0.077*	*0.977*	*0.905*	*0.910*	*0.098*	*0.705*	*0.809*	*0.716*
PhenoAge	0.13 (0.05)	0.37 (0.14)	0.04 (0.12)	0.03 (0.09)	1.07 (0.73)	−0.15(0.13)	0.16 (0.10)	0.56 (0.18)	0.14 (0.04)
*p-value*	*0.015* *	*0.007* *	*0.719*	*0.699*	*0.142*	*0.238*	*0.106*	*0.002* * ^+^	*<0.001* * ^+^
GrimAge	0.25 (0.08)	0.62 (0.22)	0.05 (0.18)	−0.14 (0.14)	2.29 (1.17)	−0.32(0.20)	0.31 (0.16)	0.86 (0.29)	0.21 (0.07)
*p-value*	*0.003* * ^+^	*0.005* * ^+^	*0.788*	*0.315*	*0.051*	*0.111*	*0.054*	*0.003* * ^+^	*0.002* * ^+^
PedBE	0.48 (0.43)	1.23 (1.12)	0.10 (0.94)	0.08 (0.69)	2.66 (6.10)	0.11 (1.06)	−0.78 (0.84)	−1.08 (1.52)	−0.24(0.35)
*p-value*	*0.262*	*0.276*	*0.919*	*0.911*	*0.662*	*0.920*	*0.357*	*0.477*	*0.500*
Wu clock	0.84 (0.39)	1.87 (1.02)	−0.46 (0.86)	−0.42 (0.63)	−2.29 (5.33)	−0.77(0.92)	−0.59 (0.74)	0.48 (1.33)	0.13 (0.31)
*p-value*	*0.032* *	*0.069*	*0.593*	*0.505*	*0.668*	*0.407*	*0.426*	*0.718*	*0.671*

SE=Standard error; BMI=Body mass index; WC=Waist circumference; BP=Blood pressure; HDL=High-density lipoprotein; HOMA-IR= Homeostasis Model Assessment of Insulin Resistance. Model adjusted for sex, socioeconomic status, smoking status, batch effects, and cell types.

* statistically significant at *p<0.05.*

+ statistically significant at Bonferroni corrected *p=0.0083(0.05/6)*.

**Table 4:** Associations between epigenetic age acceleration and cardiometabolic risk indicators at follow-up, stratified by sex.

Epigenetic age accelerations	BMI, kg/m^2^	WC, cm	Systolic BP, mmHg	Diastolic BP, mmHg	Triglycerides, mg/dL	HDL, mg/dL	Glucose, mg/dL	Insulin, mU/mL	HOMA IR
	Beta (SE)	Beta (SE)	Beta (SE)	Beta (SE)	Beta (SE)	Beta (SE)	Beta (SE)	Beta (SE)	Beta (SE)
Horvath									
Male	0.28 (0.09)	0.94 (0.26)	−0.01 (0.24)	−0.03 (0.17)	2.41 (1.36)	−0.36(0.20)	0.26 (0.17)	0.45 (0.31)	0.11 (0.07)
*p-value*	*0.003* * ^+^	*<0.001* * ^+^	*0.954*	*0.848*	*0.079*	*0.066*	*0.143*	*0.147*	*0.137*
Female	0.14 (0.11)	0.36 (0.26)	−0.03 (0.21)	−0.12 (0.15)	−1.69 (1.33)	−0.15(0.28)	−0.04 (0.20)	0.00 (0.38)	0.01 (0.09)
*p-value*	*0.194*	*0.170*	*0.898*	*0.421*	*0.207*	*0.592*	*0.828*	*1.000*	*0.944*
Skin-Blood									
Male	0.40 (0.21)	1.15 (0.58)	0.44 (0.52)	0.43 (0.38)	5.74 (3.33)	−0.96(0.48)	−0.27 (0.43)	0.79 (0.76)	0.16 (0.18)
*p-value*	*0.056*	*0.049* *	*0.400*	*0.258*	*0.087*	*0.048* *	*0.530*	*0.298*	*0.373*
Female	0.05 (0.22)	0.26 (0.56)	−0.29 (0.43)	−0.42 (0.32)	−5.00 (2.90)	−0.30 (0.61)	−0.04 (0.43)	−0.27 (0.82)	−0.01 (0.19)
*p-value*	*0.819*	*0.646*	*0.509*	*0.188*	*0.087*	*0.627*	*0.923*	*0.747*	*0.940*
PhenoAge									
Male	0.22 (0.07)	0.64 (0.21)	0.03 (0.19)	0.03 (0.14)	1.96 (1.13)	−0.13(0.16)	0.18 (0.14)	0.95 (0.25)	0.23 (0.06)
*p-value*	*0.003* * ^+^	*0.002* * ^+^	*0.875*	*0.805*	*0.083*	*0.420*	*0.227*	*<0.001* * ^+^	*<0.001* * ^+^
Female	0.07 (0.08)	0.19 (0.19)	0.10 (0.15)	0.06 (0.11)	0.47 (0.96)	−0.08(0.20)	0.12 (0.14)	0.29 (0.27)	0.08 (0.06)
*p-value*	*0.340*	*0.312*	*0.493*	*0.614*	*0.629*	*0.708*	*0.416*	*0.279*	*0.220*
GrimAge									
Male	0.27 (0.13)	0.70 (0.35)	−0.25 (0.32)	−0.41 (0.23)	2.29 (1.89)	−0.26(0.27)	0.33 (0.24)	0.80 (0.42)	0.20 (0.10)
*p-value*	*0.031* *	*0.048* *	*0.427*	*0.072*	*0.229*	*0.346*	*0.172*	*0.060*	*0.051*
Female	0.27 (0.12)	0.67 (0.30)	0.30 (0.23)	0.07 (0.17)	1.87 (1.48)	−0.36(0.31)	0.36 (0.22)	0.92 (0.41)	0.23 (0.09)
*p-value*	*0.023* *	*0.024* *	*0.199*	*0.688*	*0.210*	*0.243*	*0.102*	*0.027* *	*0.016* *
PedBE									
Male	1.46 (0.62)	3.73 (1.76)	0.02 (1.58)	−0.26 (1.15)	23.34 (9.03)	−1.33(1.33)	−0.80 (1.18)	0.92 (2.08)	0.19 (0.50)
*p-value*	*0.021* *	*0.036* *	*0.988*	*0.819*	*0.011* *	*0.317*	*0.496*	*0.658*	*0.706*
emale	−0.29 (0.60)	−0.61 (1.51)	0.11 (1.18)	0.17 (0.87)	−21.57 (7.97)	0.78 (1.69)	−0.10 (1.20)	−2.39 (2.28)	−0.46 (0.52)
*p-value*	*0.635*	*0.685*	*0.923*	*0.849*	*0.007* * ^+^	*0.645*	*0.934*	*0.296*	*0.381*
Wu clock									
Male	0.40 (0.56)	1.32 (1.57)	−0.64 (1.39)	0.47 (1.01)	−3.09 (8.16)	0.98 (1.18)	−0.88 (1.04)	1.50 (1.84)	0.29 (0.44)
*p-value*	*0.478*	*0.399*	*0.646*	*0.642*	*0.705*	*0.407*	*0.400*	*0.415*	*0.508*
Female	1.11 (0.57)	2.14 (1.44)	0.06 (1.13)	−0.73 (0.83)	−3.71 (7.26)	−2.53(1.50)	−0.29 (1.07)	0.60 (2.04)	0.25 (0.47)
*p-value*	*0.053*	*0.140*	*0.957*	*0.381*	*0.610*	*0.093*	*0.789*	*0.769*	*0.595*

SE=Standard error; BMI=Body mass index; WC=Waist circumference; BP=Blood pressure; HDL=High-density lipoprotein; HOMA-IR= Homeostasis Model Assessment of Insulin Resistance. Model adjusted for sex, socioeconomic status, smoking status, batch effects, and cell types.

* statistically significant at *p<0.05.*

+ statistically significant at Bonferroni corrected *p=0.0083(0.05/6)*.

## Appendix A. Supplementary data

Supplementary data to this article can be found online at https://doi.org/10.1016/j.numecd.2026.104574.

## Abbreviations:

ELEMENT: Early Life Exposure in Mexico to ENvironmental Toxicants
PedBE: Pediatric-Buccal-Epigenetic
HOMA-IR: Homeostatic Model Assessment of Insulin Resistance
FFQ: food frequency questionnaire
PCA: Principal component analysis
EDTA: ethylenediaminetetraacetic acid
SES: socioeconomic status
AMAI: Asociación Mexicana de Agencias de Investigación de Mercados y Opinión Pública
SE: standard error
