# Supplementary material for: Epigenetic age acceleration in adolescence: cross-sectional associations with dietary intake and prospective associations with cardiometabolic risk indicators in a Mexico City cohort
Source: Nutr Metab Cardiovasc Dis. Author manuscript; Available in PMC 2026 Jun 16. (PMC13270294; doi:10.1016/j.numecd.2026.104574)
Supplement: Supplemental Table 1 [file NIHMS2179875-supplement-Supplemental_Table_1.pdf]

Supplemental Table 2. Sensitivity analyes of fully adjusted associations* between EEAA measures and cardion

Exposure	Outcome	Beta_Estimate	Standard_Error
AgeAccelerationResidual	bmi_t2	0.158	0.07
DNAmAgeSkinBloodClockAdjAge	bmi_t2	0.182	0.16
AgeAccelPheno	bmi_t2	0.167	0.053
AgeAccelGrim	bmi_t2	0.369	0.087
ageAcc2.PedBE	bmi_t2	0.225	0.445
ageAcc2.Wu	bmi_t2	1.003	0.411
AgeAccelerationResidual	waist_t2	0.534	0.179
DNAmAgeSkinBloodClockAdjAge	waist_t2	0.59	0.409
AgeAccelPheno	waist_t2	0.501	0.135
AgeAccelGrim	waist_t2	1.063	0.219
ageAcc2.PedBE	waist_t2	0.337	1.133
ageAcc2.Wu	waist_t2	1.996	1.048
AgeAccelerationResidual	sbp_t2	-0.145	0.153
DNAmAgeSkinBloodClockAdjAge	sbp_t2	-0.358	0.347
AgeAccelPheno	sbp_t2	0.086	0.117
AgeAccelGrim	sbp_t2	0.248	0.19
ageAcc2.PedBE	sbp_t2	-0.182	0.96
ageAcc2.Wu	sbp_t2	-0.78	0.892
AgeAccelerationResidual	dbp_t2	-0.132	0.112
DNAmAgeSkinBloodClockAdjAge	dbp_t2	-0.182	0.255
AgeAccelPheno	dbp_t2	0.101	0.086
AgeAccelGrim	dbp_t2	0.051	0.14
ageAcc2.PedBE	dbp_t2	-0.114	0.705
ageAcc2.Wu	dbp_t2	-0.327	0.655
AgeAccelerationResidual	tri_t2	0.321	0.976
DNAmAgeSkinBloodClockAdjAge	tri_t2	-0.959	2.332
AgeAccelPheno	tri_t2	1.26	0.752
AgeAccelGrim	tri_t2	3.114	1.218
ageAcc2.PedBE	tri_t2	1.954	6.407
ageAcc2.Wu	tri_t2	-1.245	5.669
AgeAccelerationResidual	hdl_t2	-0.3	0.175
DNAmAgeSkinBloodClockAdjAge	hdl_t2	-0.63	0.419
AgeAccelPheno	hdl_t2	-0.188	0.136
AgeAccelGrim	hdl_t2	-0.423	0.221
ageAcc2.PedBE	hdl_t2	0.69	1.154
ageAcc2.Wu	hdl_t2	-0.94	1.02
AgeAccelerationResidual	glucose_t2	0.08	0.138
DNAmAgeSkinBloodClockAdjAge	glucose_t2	-0.185	0.33
AgeAccelPheno	glucose_t2	0.118	0.107
AgeAccelGrim	glucose_t2	0.285	0.173
ageAcc2.PedBE	glucose_t2	-0.862	0.905

ageAcc2.Wu	glucose_t2	-0.295	0.801
AgeAccelerationResidual	insulin_t2	0.241	0.252
DNAmAgeSkinBloodClockAdjAge	insulin_t2	-0.044	0.604
AgeAccelPheno	insulin_t2	0.591	0.193
AgeAccelGrim	insulin_t2	1.016	0.313
ageAcc2.PedBE	insulin_t2	-1.102	1.657
ageAcc2.Wu	insulin_t2	0.476	1.466
AgeAccelerationResidual	homa_ir_t2	0.059	0.059
DNAmAgeSkinBloodClockAdjAge	homa_ir_t2	0.008	0.14
AgeAccelPheno	homa_ir_t2	0.144	0.045
AgeAccelGrim	homa_ir_t2	0.246	0.073
ageAcc2.PedBE	homa_ir_t2	-0.248	0.385
ageAcc2.Wu	homa_ir_t2	0.161	0.341

*Models include adjustment for batch effects, cell types, maternal education, MVPA, sedentary behavor, height

etabolic outcomes

P_Value
0.025
0.256
0.002
0
0.613
0.015
0.003
0.149
0
0
0.766
0.058
0.343
0.302
0.46
0.193
0.85
0.383
0.241
0.475
0.237
0.713
0.871
0.618
0.742
0.681
0.095
0.011
0.761
0.826
0.087
0.133
0.168
0.056
0.55
0.358
0.562
0.575
0.271
0.102
0.342

0.713
0.341
0.942
0.002
0.001
0.506
0.746
0.316
0.954
0.001
0.001
0.52
0.638

[illegible], pubertal status, age difference, and alcohol intake behavior
