# Supplementary material for: Epigenetic age acceleration in adolescence: cross-sectional associations with dietary intake and prospective associations with cardiometabolic risk indicators in a Mexico City cohort
Source: Nutr Metab Cardiovasc Dis. Author manuscript; Available in PMC 2026 Jun 16. (PMC13270294; doi:10.1016/j.numecd.2026.104574)
Supplement: Supplemental Table 2 [file NIHMS2179875-supplement-Supplemental_Table_2.pdf]

Supplemental Table 1. Correlations between EEAA measures and potential confounders or adjustment variables

		AgeAccelerationResidual	DNAmAgeSkinBloodClockAdjAge	AgeAccelPheno	AgeAccelGrim	ageAcc2.PedBE	ageAcc2.Wu
Batch effects	**PC1**	-0.08	-0.05	0.11	-0.08	0.14	0.06
	**PC2**	0.22	0.17	-0.08	0.18	-0.35	-0.10
	**PC3**	-0.30	0.04	-0.04	-0.05	-0.08	0.25
	**PC4**	-0.06	0.01	0.21	-0.01	0.02	-0.17
	**PC5**	0.39	0.09	0.02	0.27	-0.07	-0.23
Cell types	**CD8T**	0.11	0.14	-0.32	-0.24	0.04	0.19
	**CD4T**	0.00	-0.10	-0.48	-0.18	-0.34	-0.10
	**NK**	0.17	0.21	-0.02	-0.02	-0.05	0.19
	**Bcell**	0.12	-0.08	-0.36	-0.17	-0.22	0.07
	**Mono**	-0.16	-0.08	0.29	0.17	0.09	-0.02
	**Gran**	-0.19	-0.11	0.45	0.20	0.23	-0.12
	**PlasmaBlast**	-0.07	-0.06	0.41	0.20	0.19	-0.04
	**CD8pCD28nCD45RAn**	0.15	0.17	0.23	0.16	0.17	0.17
	**CD8.naive**	0.09	-0.07	-0.47	-0.06	-0.37	-0.19
	**CD4.naive**	-0.02	-0.03	-0.41	-0.15	-0.23	-0.11
Lifestyle confounders	**ses**	-0.02	0.02	-0.01	0.00	-0.02	0.04
	**maternal education**	-0.03	-0.06	-0.07	-0.06	-0.08	-0.12
	**smoking**	0.00	0.01	-0.09	0.01	-0.07	0.00
	**mvpa**	0.00	0.05	-0.03	-0.04	-0.04	0.00
	**Age_yrs**	-0.02	0.03	0.08	-0.08	0.15	-0.05
	**sex**	-0.18	0.03	0.14	-0.06	-0.06	-0.07
EEAA measures	**AgeAccelerationResidual**	1.00	0.48	0.25	0.34	0.11	0.02
	**DNAmAgeSkinBloodClockAdjAge**	0.48	1.00	0.28	0.22	0.31	0.33
	**AgeAccelPheno**	0.25	0.28	1.00	0.37	0.31	0.10
	**AgeAccelGrim**	0.34	0.22	0.37	1.00	-0.07	-0.09
	**ageAcc2.PedBE**	0.11	0.31	0.31	-0.07	1.00	0.19
	**ageAcc2.Wu**	0.02	0.33	0.10	-0.09	0.19	1.00
